# Age-dependent gene expression in the inner ear of big brown bats (*Eptesicus fuscus*)

**DOI:** 10.1371/journal.pone.0186667

**Published:** 2017-10-26

**Authors:** Beatrice Mao, Cynthia F. Moss, Gerald S. Wilkinson

**Affiliations:** 1 Department of Biology, College of Computer, Mathematical, and Natural Sciences, University of Maryland, College Park, Maryland, United States of America; 2 Department of Psychological and Brain Sciences, Zanvyl Krieger School of Arts and Sciences, Johns Hopkins University, Baltimore, Maryland, United States of America; 3 The Solomon H. Snyder Department of Neuroscience, Johns Hopkins University School of Medicine, Johns Hopkins University, Baltimore, Maryland, United States of America; Texas A&M University College Station, UNITED STATES

## Abstract

For echolocating bats, hearing is essential for survival. Specializations for detecting and processing high frequency sounds are apparent throughout their auditory systems. Recent studies on echolocating mammals have reported evidence of parallel evolution in some hearing-related genes in which distantly related groups of echolocating animals (bats and toothed whales), cluster together in gene trees due to apparent amino acid convergence. However, molecular adaptations can occur not only in coding sequences, but also in the regulation of gene expression. The aim of this study was to examine the expression of hearing-related genes in the inner ear of developing big brown bats, *Eptesicus fuscus*, during the period in which echolocation vocalizations increase dramatically in frequency. We found that seven genes were significantly upregulated in juveniles relative to adults, and that the expression of four genes through development correlated with estimated age. Compared to available data for mice, it appears that expression of some hearing genes is extended in juvenile bats. These results are consistent with a prolonged growth period required to develop larger cochlea relative to body size, a later maturation of high frequency hearing, and a greater dependence on high frequency hearing in echolocating bats.

## Introduction

Echolocating bats have among the highest frequency hearing in the animal kingdom [1]. While high frequency hearing confers a survival benefit to many animals, it is essential for the survival of bats, because they rely on echolocation to avoid obstacles, obtain food, and find roosts and conspecifics. High frequencies also allow bats to control the directionality of calls [2], [3], determine distance to targets [4], reject non-target echo clutter [5], and resolve fine spatial details such as shape, size, and texture [6–8]. Furthermore, bats are exceptionally long-lived for their size, with individuals of some species living more than 30 years [9]. The need for echolocation throughout life suggests that the ability to hear high frequencies without severe age-related deterioration may have been under positive selection in echolocating bats. This stands in contrast with the occurrence of age-related hearing loss (presbycusis) in humans, which has been estimated to be 40% among those over 70 [10].

The importance of hearing to echolocators has been illustrated by a number of recent studies examining the molecular evolution of genes involved in hearing in bats. Several genes known from human and mouse studies to be crucial for normal hearing, such as transmembrane channel-like 1 (*Tmc1*) and *Prestin/SLC26A5*, exhibit convergence between the two distantly related groups of echolocating bats, or even between echolocating bats and whales, such that gene trees sometimes group echolocators together to the exclusion of non-echolocators [11–17]. While the results of these studies are compelling, the amount or timing of gene expression may also contribute to different phenotypes without requiring changes in coding sequence. Recent studies have shown that changes in gene regulation can influence the physical differences between bats and other mammals: transgenic mice possessing bat limb enhancers exhibit prolonged expression of limb elongation genes [18] and develop significantly longer limbs than control mice [19].

The big brown bat (*Eptesicus fuscus*) is an insectivore that hunts in edge spaces between open and cluttered environments [20]. This behavior requires the disambiguation of cascades of echoes from multiple objects into separate percepts [21–22], which must occur quickly enough to inform motor decisions in flight. Because echolocation and flight are critical for a young bat’s survival, the development of hearing occurs concurrently with echolocation calls and the motor skills involved in flight [23–28]. The echolocation calls of juvenile big brown bats undergo significant changes between birth and three weeks of age, becoming shorter in duration and higher in frequency [26–29]. These changes in echolocation call frequencies likely coincide with changes in their hearing, because the frequency place map of the cochlea changes as it matures, with higher frequency hearing developing later [30, 31]. Additionally, the call frequencies of five species of bats were lower in the first year of life than later in adulthood, suggesting that fine-tuning of echolocation calls may occur well after the development of hearing is complete (summarized in [32]).

Because of their dependence on hearing for survival and their relatively well-developed auditory systems, echolocating bats provide a valuable opportunity to examine postnatal hearing development in an auditory specialist. Laryngeally echolocating bats possess larger cochlea [33] relative to basicranial width than non-echolocating or non-laryngeally echolocating bats [34]. Bats using constant-frequency calls also exhibit overrepresentation of dominant call frequencies in basilar membrane (BM) dimensions and spiral ganglion density [35], and extremely short hair cells and stereocilia [36]. A recent study showed that echolocating bats sustain a high prenatal cochlear growth rate throughout development compared to non-echolocating bats and other mammals [37], but which genes change expression during bat cochlear development is unknown. Here, we report on the expression of selected hearing-related genes in the inner ears of young big brown bats over a two-week period during which their calls rapidly increase in frequency, becoming more similar to adult echolocation calls [26–29]. Because these pronounced frequency shifts in vocalizations have been reported to coincide with frequency shifts in hearing in several bat species (e.g., [24, 38, 39]), examining gene expression during this period may provide insight into the regulatory changes associated with the development of high frequency hearing.

## Materials and methods

### Subjects and sample preparation

Pregnant female *Eptesicus fuscus* were captured in the wild under a permit from the Maryland Department of Natural Resources. All twelve juvenile subjects were born in captivity. Because they were group-housed and cluster together, exact dates of birth could not be directly recorded. Instead, forearm length was measured with calipers and used to estimate age [40]. Forearm length is a more accurate age estimator than mass for big brown bats, and results from formulae relating forearm length to age do not differ between wild and captive bats [28]. Estimated ages ranged from postnatal day (PND) 9 to 19. Juveniles were weighed, anesthetized with isoflurane and euthanized via decapitation. All procedures were in accordance with the National Institutes of Health’s *Guide for the Care and Use of Laboratory Animals*, and were approved by the Johns Hopkins University Institutional Animal Care and Use Committee (protocol BA14A111). Samples were also obtained from two adult individuals under a protocol approved by the University of Maryland Institutional Animal Care and Use Committee (R-13-76).

Inner ear samples, consisting of the entire otic capsule (both cochleae and vestibular organs), were collected immediately post-mortem and placed into liquid nitrogen prior to storage at -80°C until extraction. Both left and right cochleae from an individual were pooled and processed together. Samples were homogenized with a mortar and pestle while submerged in liquid nitrogen. RNA extraction was performed using a mirVana kit (Ambion), with added proteinase K (Sigma Aldrich) to improve yield [41]. All samples were treated with TURBO DNA-free DNAse (Ambion) and cleaned with isopropanol and ethanol. Sample quality was checked on a Nanodrop spectrophotometer and reverse transcribed with M-MLV (Thermo Fisher) using a 50/50 mix of oligo-dT and random primers to lower the risk of bias or truncated transcripts associated with a single priming method [42, 43].

### Gene selection and primer design

Candidate genes were selected based on one or more of the following criteria: upregulated in an echolocating bat vs. a non-echolocating bat (e.g., [44]); upregulated in an adult mouse relative to juvenile mouse (e.g., [45]); expressed in mid- to late- development (e.g., [46]); evidence of parallel or convergent evolution between echolocating bats and whales (e.g., [16]); evidence of parallel or convergent evolution between distantly related echolocating bats (e.g., [16]); or involved in formation of essential cochlear structures (e.g., [47]; Table 1). For each gene, all available mRNA transcripts from *Eptesicus fuscus* and all bats of the genus *Myotis* (another genus in the same family, Vespertilionidae), were downloaded from GenBank (NCBI) and aligned using Clustal Omega (EMBL-EBI). Sequences from *Myotis* spp. were included in order to reduce the risk of designing primers in regions with polymorphic sites. All primer pairs were designed within the same exon to permit preliminary testing on genomic DNA.

**Table 1:** Criteria for inclusion and other relevant information for genes included in this study, and references. In the “Criteria for inclusion column,” letter codes mean the following: A, upregulated in an echolocating bat vs. a non-echolocating bat; B, upregulated in an adult mouse relative to juvenile mouse; C, expressed in mid- to late- development; D, exhibits signs of parallel or convergent evolution between echolocating bats and whales; E, exhibits signs of parallel or convergent evolution between distantly related echolocating bats; F, participates in forming essential cochlear structures. ^a^Mutations in *Gjb6* may cause hearing loss by inducing a downregulation of *Gjb2*. *Gjb6* appears not be critical for hearing, unlike *Gjb2* (see [64]).

Gene symbol	Full name of gene	Criteria for inclusion	Location of gene product	Morphological effects of deletion or mutation in mouse models	Associated with human deafness (and loci if applicable)	Sources
Bmp7	Bone morphogenic protein 7	F	throughout cochlear duct	loss of position-specific sensory cell morphology consistent with loss of tonotopy	yes	[48, 49]
Ceacam16	Carcinoembryonic antigen-related cell adhesion molecule 16	A, F	tallest OHC stereocilia tips; TM	disruption of normal striated-sheet matrix of TM, Hensen’s stripe absent	DFNA4	[44, 50–53]
Col11A2	Collagen type XI alpha 2 chain	A	TM, cartilaginous otic capsule, spiral limbus, lateral wall, cristae ampullaris	enlarged TM containing disorganized collagen fibrils; reduced density of radial collagen fibers in the TM	DFNA13; DFNB53	[44, 54–56]
GFAP	Glial fibrillary acidic protein	B	supporting cells, Schwann cells in SG and osseous spiral lamina	greater loss of OHCs after noise exposure		[45, 57, 58]
Gjb2	Gap junction protein beta 2	AF	gap junctions of supporting cells	severe degeneration of the organ of Corti and SGN loss	DFNB1	[44, 59–61]
Gjb6 ^a^	Gap junction beta protein 6	A, F	gap junctions of supporting cells	missing OHCs	DFNB1; DFNA3	[44, 62–65]
LOXHD1	Lipoxygenase homology domains 1	A, B	cochlear and vestibular hair cell stereocilia	fused stereocilia and ruffled apical cell surface at cochlear base, leading to eventual hair cell and SGN loss	DFNB77	[44, 66]
Pou3F4	POU class 3 transcription factor 4	A	throughout otic capsule	radial bundle defasciculation; abnormal gap junctions; malformed stapes footplate; reduced cochlear coiling; other abnormalities	DFNX2	[44, 67–69]
Pou4f3	POU class 4 transcription factor 3	C	nuclei of cochlear and vestibular hair cells	loss of auditory and vestibular hair cells; failure of differentiated hair cells to develop stereociliary bundles; loss of spiral and vestibular ganglion neurons	DFNA15	[46, 70, 71]
Tmc1	Transmembrane channel-like 1	A, D, E, F	MET channels of hair cells	none	DFNA36; DFNB7; DFNB11	[16, 44, 47, 72–74]
Tmc2	Transmembrane channel-like 2	F	MET channels of hair cells	none		[72–74]
Tspan1	Tetraspanin 1	B	in zebrafish, rostral mantle cells within neuromasts			[45, 75]
Ush1C	USH1 protein network component harmonin	A, B, C, F	Upper tip link density of stereocilia bundles; cochlear and vestibular neurosensory epithelia	splayed hair cell bundles; progressive degeneration of hair cells	DFNB18	[44, 76–80]

To identify exons in an *Eptesicus fuscus* transcript, exonic regions of the *Myotis lucifugus* transcript, as identified in Ensembl, were blasted against the transcript for *Eptesicus fuscus*. If the *Myotis* transcript was not available in Ensembl, the mouse (*Mus musculus*) transcript was used instead. If the exonic region was conserved among *Eptesicus* and *Myotis* spp., it was entered into Primer-BLAST (NCBI). Potential primer pairs were checked for specificity against *Eptesicus fuscus* RefSeq data, potential for cross- and self-dimerization, and potential to form hairpins using Beacon Designer (Premier Biosoft). Only primers that were 100% conserved across all known transcripts from *Eptesicus* and *Myotis* spp. were used for quantitative PCR. Primer sequences are given in Table 2.

**Table 2:** Primers used to amplify *Eptesicus fuscus* cDNA and calculated efficiencies based on dilution series. Efficiencies greater than 100% typically indicate the presence of inhibitors, the effects of which decrease at lower dilutions.

Gene	Forward primer	Reverse primer	Efficiency (%)
Bmp7	CCTACAAGGCGGTCTTCAGC	CGTCGGTGAGGAAGTGGCTA	102.2
Ceacam16	ACATCGTAAGCACAGGCGAC	CTGAAGGATGTAGGTGCCCG	102.6
Col11A2	CGAAGTGCTCGTCCAGTGTTG	ATCCAGGATACGGGCACCAAA	101.6
GAPDH	GGGCTGCCCAGAACATCATC	GCTCAGGGATGACCTTGCC	109.4
GFAP	CACCGGCTTCAAGGAGACAC	TTCTCGATGTAGCTGGCGAAG	101.4
Gjb2	CAGAAGGTCCGAATTGAAGGGT	AAGATGACCCGGAAGAAGATGC	108.0
Gjb6	TTCATCGGGGGTGTGAACAAA	CACGAGGATCATGACACGGAAG	95.6
LoxHD1	CGAGATCGTCATAGAAACGGGC	TCTTTGGATCGGTTCTTCCTGC	102.5
Pou3f4	AGCGATCTAGGCTCTCACCA	CATCCGAGGTTGGTGTCTCC	111.0
Pou4f3	TGGATATCGTCTCCCACGGC	TGGTATGGTAGGTGGCGTCG	108.3
Tmc1	CTCATCTTTTGGGCTGTGAAG	CCCAAGGGTGTCAGGATCTT	102.0
Tmc2	CAGGACTGGTGGGCATCAAC	GTTGGATCGGGAGGCTTTGA	107.2
Tspan1	GTGCTCTTGGCTCTCGGTTT	AGGGCACACTTGTTCTCAGTG	109.9
Ush1C	GCTGGAAGAGGTGAGGCAG	CTTGTTGGACTCCATCGCCA	103.9

Five-point dilution series (1:3 or 1:4) were performed for each gene and only primer pairs with efficiencies greater than 90% after exclusion of non-linear dilutions (typically at the highest or lowest concentration of template) were selected for use. Post-amplification melt curves were checked to ensure each product consisted of a single, narrow peak, and gel electrophoresis was performed for each amplicon to ensure a single product of correct size was produced during amplification.

### qPCR and data analysis

For each primer pair, 20 μL reactions were prepared for each of the samples in triplicate using SYBR Select Master Mix (Thermo Fisher). Glyceraldehyde-3-phosphate dehydrogenase (*GAPDH*) was included as a reference gene on each 96-well plate. Fluorescence was measured using a Roche 480 Lightcycler and melt curves were measured immediately after the completion of all amplification cycles. Technical replicates that reached threshold two or more cycles earlier or later than the other two replicates were excluded from analyses.

For each sample-primer combination on a given plate, the comparative C_T_ method [81] was used to calculate relative expression. Briefly, delta C_T_ was calculated as the average threshold cycle of replicates from the gene of interest minus the average threshold cycle of the *GAPDH* replicates. To control for any batch effects, delta C_T_ values were adjusted by the difference in mean delta C_T_ between batches for each gene. Delta C_T_ values were then normalized by subtracting the average delta C_T_ for all juvenile samples for a given gene (yielding delta-delta C_T)_. Fold expression was calculated as the efficiency-adjusted amplification factor raised to the negative delta-delta C_T._ Average C_T_ and calculated fold expression values are given in S1 Table.

We performed t-tests to determine whether the mean adjusted fold expression values of juveniles differed from adults for 13 genes. We also fitted least squares regression lines between estimated age and adjusted fold change to identify genes that exhibited age-dependent expression. All statistical analyses were performed in JMP 13.0.0 (SAS Institute). Figures were generated in JMP and MATLAB R2015a (The Mathworks).

## Results

### Adult vs. juvenile expression

**Fig 1:**
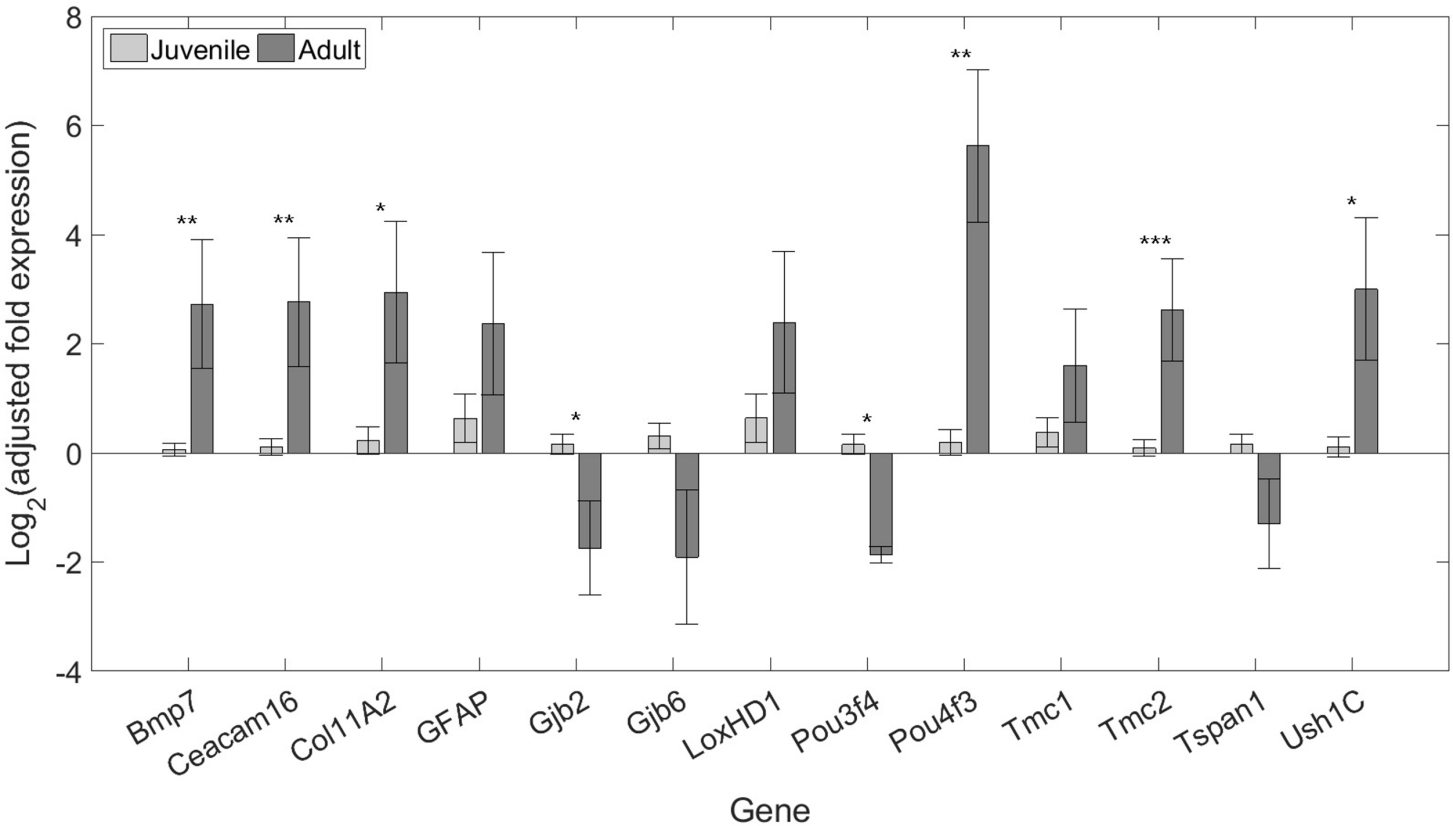
Log_2_-scaled means and standard errors of adult and juvenile expression relative to *GAPDH*. Values were adjusted to remove the effect of batch and normalized to average juvenile expression (see Materials and Methods). Juvenile data are shown in light grey, and adult data are shown in dark grey. Asterisks denote level of significance of associated t-tests (see Table 3; *p≤0.05, **p≤0.01, ***p≤0.005).

Of the 13 genes tested, eight exhibited differential expression between juveniles and adults (Table 3; Fig 1). Expression was higher in adults for six genes—bone morphogenic protein 7 (*Bmp7*), carcinoembryonic antigen-related cell adhesion molecule 16 (*Ceacam16*), collagen type XI alpha 2 chain (*Col11A2*), POU class 4 transcription factor 3 (*Pou4f3*), transmembrane channel-like 2 (*Tmc2*), and USH1 protein network component harmonin (*Ush1C*), and higher in juveniles for the remaining two genes—gap junction protein beta 2 (*Gjb2*) and POU class 3 transcription factor 4 (*Pou3f4*).

**Table 3:** Results of two-sided t-tests performed on adjusted fold change between adults and juveniles (left) and bivariate fits of adjusted fold change by estimated age (right). For all t-tests, there were 13 degrees of freedom, and bivariate fits had 11 degrees of freedom. Fold change values were adjusted to the mean of all juvenile samples and also to differences in mean juvenile expression between batches (see Materials and Methods). Asterisks denote level of significance (*p≤0.05, **p≤0.01, ***p≤0.005).

	Adult vs. juvenile t-test				Age vs. adjusted fold change bivariate fit		
	t Ratio	p Value	Mean ± SE, adult	Mean ± SE, juvenile	F ratio	p Value	Adjusted R^2^
**Bmp7**	-3.25**	<0.01	6.60 ± 5.44	1.04 ± 0.09	1.75	0.22	0.06
**Ceacam16**	-3.22**	<0.01	6.79 ± 5.61	1.08 ± 0.12	1.88	0.20	0.07
**Col11A2**	-2.92*	0.01	7.70 ± 6.98	1.17 ± 0.20	0.84	0.38	-0.02
**GFAP**	-1.90	0.08	5.16 ± 4.68	1.55 ± 0.48	0.39	0.55	-0.06
**Gjb2**	2.21*	0.05	0.30 ± 0.18	1.12 ± 0.14	14.85***	<0.01	0.56
**Gjb6**	1.89	0.08	0.27 ± 0.23	1.25 ± 0.20	18.62***	<0.01	0.62
**LoxHD1**	-1.93	0.08	5.24 ± 4.76	1.56 ± 0.48	0.32	0.58	-0.07
**Pou3f4**	2.31*	0.04	0.28 ± 0.03	1.11 ± 0.14	7.32*	0.02	0.37
**Pou4f3**	-3.21**	<0.01	49.44 ± 48.20	1.15 ± 0.19	1.02	0.34	0
**Tmc1**	-1.88	0.08	3.03 ± 2.18	1.30 ± 0.24	5.82*	0.04	0.31
**Tmc2**	-3.97***	<0.01	6.18 ± 4.04	1.06 ± 0.11	1.14	0.31	0.01
**Tspan1**	1.98	0.07	0.41 ± 0.23	1.12 ± 0.14	3.75	0.08	0.2
**Ush1C**	-3.01*	0.01	7.98 ± 7.27	1.08 ± 0.14	1.49	0.25	0.04

### Age-related gene expression

**Fig 2:**
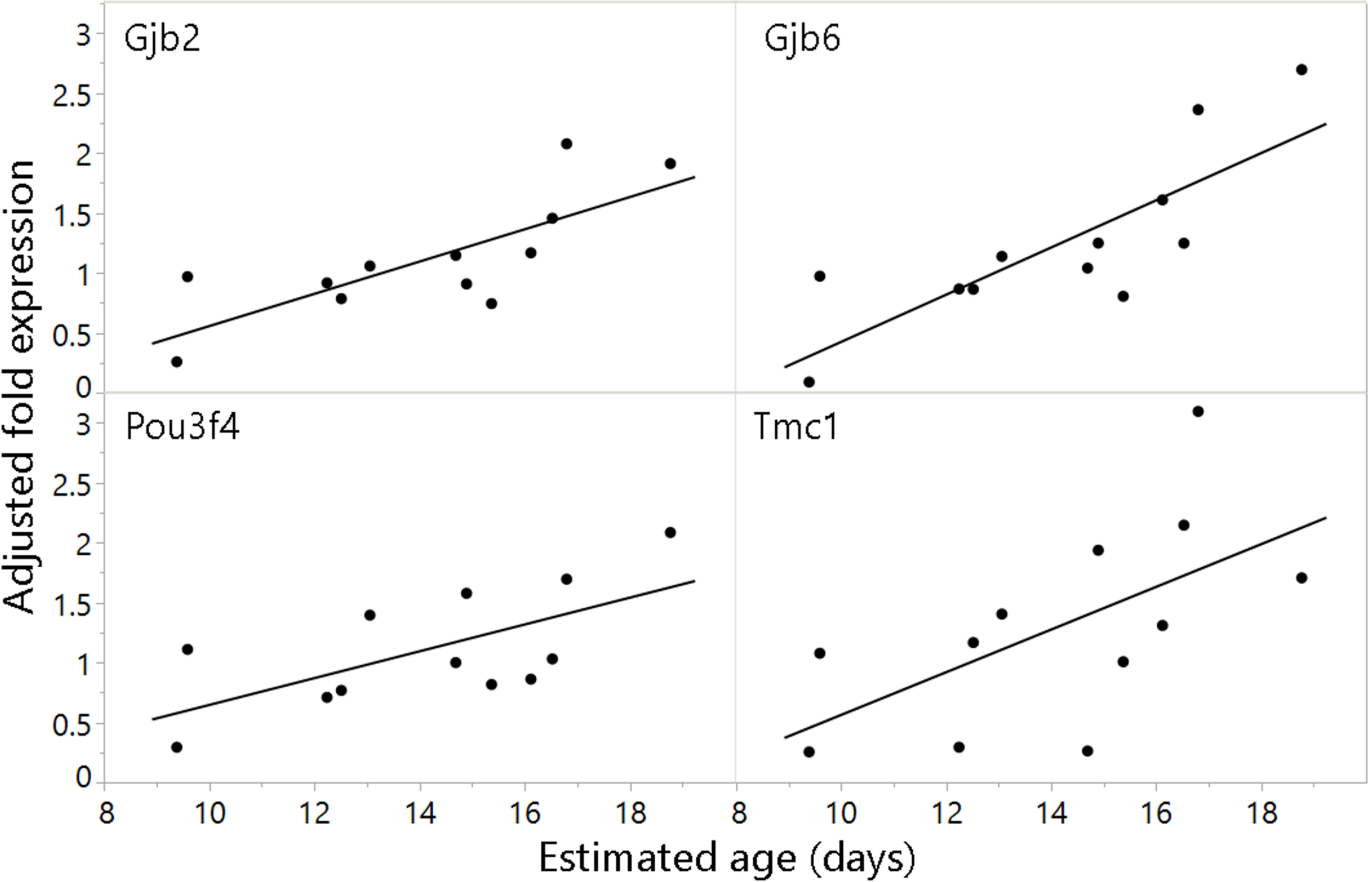
Genes for which the relationship between adjusted fold change and estimated age was significant for juvenile bats. Values were normalized to average juvenile expression and adjusted to remove the effect of batch (see Materials and Methods). Asterisks denote level of significance of associated t-tests (see Table 3; *p≤0.05, **p≤0.01, ***p≤0.005).

Linear fits of adjusted fold change to estimated age revealed that juvenile age over a two-week period predicted expression for four genes: POU class 3 transcription factor 4 (*Pou3f4*), transmembrane channel-like 1 (*Tmc1*), and gap junction protein beta 2 (*Gjb2*) and 6 (*Gjb6*; Table 3; Fig 2).

## Discussion

### Adult vs. juvenile expression

We found significant differences between juvenile and adult bat in inner ear expression of eight genes. The most significantly upregulated gene in adults was *Tmc2* (Fig 1). *Tmc1* and *Tmc2* are expressed in the cochlea and vestibular system [72, 74], and encode components of the mechanoelectrotransduction (MET) channels of hair cells [73]. Their protein products may form heteromeric assemblies that confer different electrophysiological properties to hair cells along the BM [73]. Despite functional redundancy between *Tmc1* and *Tmc2*, mice with a targeted deletion of *Tmc1* are deaf because *Tmc2* does not persist in the cochlea beyond early postnatal ages [72]. In the utricle, *Tmc1* and *Tmc2* expression continues through the first few postnatal weeks [72]. These observations in postnatal mice suggest that continued *Tmc2* expression into adulthood in bats may be restricted to the balance organs.

*Bmp7*, *Ceacam16*, *Col11A2*, and *Ush1C* were also upregulated in adults relative to juveniles (Fig 1). *Bmp7* is expressed in a gradient along the basilar papilla, and disruption of this gradient results in loss of tonotopy and morphological changes in sensory cells [49]. While we found that it was upregulated in adult bats, another study reported that *Bmp7* is downregulated in the cochlear sensory epithelia of P60 mice relative to P1 mice [82]. *Ush1C* encodes a protein, harmonin, that is a component of upper tip-link densities of stereocilia bundles [78]. Mutations in *Ush1C* are associated with Usher syndrome type 1C in humans [76], and mouse mutants exhibit splayed stereocilia bundles and progressive loss of hair cells and spiral ganglion neurons [77]. Cochlear expression of *Ush1C* drops prior to birth and then increases into adulthood in mice [79] and is similarly expressed at higher levels in adult than juvenile bats (Table 3; Fig 1).

Both *Ceacam16* and *Col11A2* encode proteins that are components of the tectorial membrane (TM), and their deletion disrupts TM structure [53, 56], resulting in hearing loss [52, 54]. The TM acts as an inertial mass which allows the outer hair cells (OHCs) to amplify BM motion [83]. Reducing its mass by deleting *Tectb* improved the frequency selectivity of the BM and neural response at high frequencies [84]. *Ceacam16* may stabilize interactions between TM glycoproteins, such that cochlear amplification becomes unstable without it [53]. The upregulation of *Col11A2* and *Ceacam16* may, therefore, result in a TM structure which allows bat hair cells to effectively amplify high frequency sounds.

*Pou4f3* showed the greatest difference in expression between age groups (Fig 1). *Pou4f3* is a transcription factor implicated in progressive non-syndromic hearing loss in humans [71]. Mice lacking *Pou4f3* fail to develop stereocilia bundles [46], resulting in the loss of hair cells and spiral ganglion neurons [70]. *Pou4f3* is expressed into adulthood in mice [46, 70, 85] but is downregulated in the P60 mouse cochlea compared to P1 [82]. Taken together, the upregulation of *Tmc2*, *Bmp7*, *Ush1C*, *Ceacam16*, *Col11A2*, and *Pou4f3* in adult big brown bats may reflect continued development or maturation of the inner ear that continues beyond the time point at which bats can fly and produce adult-like echolocation calls. The two genes that were significantly upregulated in juveniles relative to adult bats, *Gjb2* and *Pou3f4*, are discussed in further detail in the next section, as their expression also correlated with juvenile age.

### Age-related gene expression

Four genes were significantly upregulated with age in juvenile bats. Of these, *Gjb2*, *Gjb6*, and *Pou3f4* were downregulated in adult bats relative to juvenile bats, perhaps because their roles in inner ear development were complete (Table 3; Figs 1 and 2). The expression of the fourth gene, *Tmc1*, did not differ significantly between juveniles and adults, although standard errors for adult samples were high due in part to small sample size (Fig 1). While levels of the protein products (Cx26 and Cx30) of *Gjb2* and *Gjb6* saturate at P15 in the mouse cochlea [86], we found that *Gjb2* and *Gjb6* expression increased through the third postnatal week in the inner ears of bats. In an earlier report, these genes were significantly upregulated in the inner ears of an echolocating bat (*Myotis ricketti*) compared to a non-echolocating bat (*Cynopterus sphinx*) [44]. *Gjb2* appears critical for cochlear function and is implicated in the most common form of congenital deafness in humans [59, 87]. *Gjb6* has also been linked to human deafness [62], although the deleterious effects of *Gjb6* knockdown in mice are less severe than those of *Gjb2* and may be partly caused by associated downregulation of *Gjb2* [64, 88].

The upregulation of *Gjb2* and *Gjb6* may reflect greater numbers of gap junctions in the bat cochlea. Both genes may participate in the recycling of potassium, the major charge carrier in transduction (reviewed in [89]). Conditional knockdown of *Gjb2* in early postnatal mice impaired OHC amplification and high frequency hearing [90], consistent with gap junction conductivity enabling OHCs to respond to higher frequencies [91–93]. The continued expression of Gjb2 and Gjb6 may also result from prolonged development of the cochleae, which are relatively large in echolocating bats [33, 34]. A recent paper showed that the relative median prenatal growth rate of echolocating bats’ cochleae was approximately two and four times larger, respectively, than that of non-echolocating mammals and non-laryngeally echolocating bats [37].

*Gjb2* and *Gjb6* upregulation may provide some protection against hearing loss in echolocating bats, which depend on hearing throughout their long lives. Conditional knockdown of *Gjb2* in mice at P18 resulted in greater susceptibility to noise-induced hearing loss at P30 and P45 [94], and mice lacking *Gjb6* exhibited abnormal epithelial repair after hair cell loss and reduced intercellular communication between supporting cells [95]. Cx26 and Cx30 may be targets of oxidative damage, contributing to age-related and noise-induced hearing loss [96]. The increase of *Gjb2* and *Gjb6* expression during juvenile development in bats may, therefore, be associated with a system of gap junctions that facilitates cochlear protection or repair. After an hour of broadband noise exposure at 152 dB SPL, adult big brown bats showed no significant threshold shifts [97, 98], increase in errors, or changes in echolocation behavior when flying through a cluttered corridor [99]. Additionally, bat echolocation calls can be as intense as 140 dB, although they last only milliseconds [100], and it is unclear whether wild bats encounter sounds that could damage their hearing.

*Pou3f4* is a transcription factor that has been implicated in X-linked non-syndromic deafness [67]. *Pou3f4* mouse mutants exhibit audiological and balance impairments, reduced coiling of the cochlea [68], and defects in gap junctions [101]. Deletion of *Pou3f4* from otic mesenchyme causes defasciculation of spiral ganglion neurons [69], which could disrupt coordination of hair cell and neuronal frequencies [102]. These studies suggest that the continued upregulation of *Pou3f4* in the developing bat inner ear may be linked to cochlear elongation and functional organization. One report did not find evidence of positive selection on *Pou3f4* among echolocating bats [103], suggesting that change in expression, rather than sequence, has been more important in bats.

*Tmc1* encodes a MET channel protein [73] that localizes to the tip-links of stereocilia [74] and which is essential for mechanotransduction in cochlear hair cells [72]. Reports of its postnatal expression pattern conflict: one study found a slight increase, then decrease in *Tmc1* expression in the inner ear of mice from P9 to P19, with a net decrease of approximately 8% over the period [47]. Another study reported a 2-fold increase between P9 and P19 in the utricle and a much greater increase over the same time period in the apex of the cochlea [72]. The increase in *Tmc1* we observed in developing big brown bats is consistent with the latter study, and with a transcriptomic comparison of the inner ears of bats which showed that 18 hearing-related genes were upregulated in an echolocating bat compared to a non-echolocating bat, including *Tmc1*, which was also upregulated in echolocating bats relative to mice and rats [44].

Although the nature of our samples (entire inner ears) did not permit examination of gene expression specifically in the cochlea or its basal, high frequency region, the upregulation of *Tmc1* could reflect a greater number of MET channels per hair cell, which might increase sensitivity to high frequencies by strengthening the influx of calcium and reducing the adaptation time of hair cells (reviewed in [104]). In midshipman fish (*Porichthys notatus*), fluctuations in the expression of a calcium-activated potassium (BK) channel conferred greater hearing sensitivity during the breeding season [105], and knockdown of BK channel genes increased thresholds in zebrafish larvae [106]. Alternatively, bat MET channels may contain more Tmc1 subunits. Because mouse hair cells expressing only wildtype *Tmc1* had faster adaptation times than those expressing only *Tmc2* or only a *Tmc1* mutant [73], MET channels incorporating more Tmc1 subunits might respond better at high frequencies.

Although only a small set of genes were examined in this study, and we did not manipulate gene expression directly and monitor subsequent phenotypic effects, this study provides the first insight into the developmental expression of hearing genes in echolocating animals. Without separation of the cochlea from the vestibular organs, it is not possible to ascribe expression differences to one section of the inner ear or the other. However, *Tmc1* and *Gjb2* mouse mutants exhibit hearing loss without vestibular dysfunction, illustrating their greater importance for audition [47, 60]. Furthermore, hearing genes exhibiting various degrees of convergence between echolocating bats and whales have been implicated in human deafness [11–17], as have most of the genes we identified as being significantly upregulated with age in big brown bats. In particular, *Tmc1* exhibits both sequence convergence [16] and upregulation ([44], this report) in echolocators, suggesting that in some cases selection may act on both coding sequence and gene regulation to confer improved hearing in echolocating mammals.

## Supporting information

S1 Table Average C_T_ and calculated fold expression values for all individuals and genes. (XLSX)
